# The Effect of Strength Training on Vastus Lateralis’ Stiffness: An Ultrasound Quasi-Static Elastography Study

**DOI:** 10.3390/ijerph17124381

**Published:** 2020-06-18

**Authors:** Rute Santos, Maria João Valamatos, Pedro Mil-Homens, Paulo Armada-da-Silva

**Affiliations:** 1 Faculty of Human Motricity, University of Lisbon, 1499-002 Estrada da Costa, Portugal; mjvalamatos@fmh.ulisboa.pt (M.J.V.); pmil@fmh.ulisboa.pt (P.M.-H.); parmada@fmh.ulisboa.pt (P.A.-d.-S.); 2 Department of Medical Imaging and Radiotherapy, Coimbra Health School, Polytechnic Institute of Coimbra, 3046-854 Coimbra, Portugal

**Keywords:** quantitative ultrasound, elastography, training program, musculoskeletal

## Abstract

Ultrasound imaging allows the evaluation of musculoskeletal morphology and function. Ultrasound elastography can also offer semi-quantitative and/or quantitative assessment of tissue stiffness providing relevant information about adaptations of skeletal muscle mechanical properties. In this study we aimed to explore the feasibility of elastography ultrasound imaging in assessing the effect of strength training on vastus lateralis stiffness. Twenty-eight young male adults were separated in a control (*n* = 9) and strength-training (*n* = 20) groups. The strength-training group completed 15 weeks of either concentric (*n* = 10) or eccentric (*n* = 10) isokinetic training of the knee extensors. Ultrasound scans of the vastus lateralis for quasi-static elastography were collected. All image acquisitions and measurements were done by the same experienced sonographer. After 15 weeks, knee maximal extension isometric torque increased in the strength-training groups. After strength training, there was a decrease in the amount of red pixels in vastus lateralis region of interest [F_(1,18)_ = 25.490; *p* < 0.001; η^2^ = 0.586], whereas the amount of green and blue pixels increased F_(1,18)_ = 17.179; *p* < 0.01; η^2^ = 0.488; F_(1,18)_ = 6.522; *p* < 0.05; η^2^ = 0.266], suggesting higher vastus lateralis stiffness. We conclude that concentric and eccentric strength training increases skeletal muscle stiffness, which can be evaluated by quasi-static elastography. Ultrasound elastography is suitable for non-invasive evaluation of skeletal muscle functional adaptations, which can be of importance for sports medicine and in designing optimal training and rehabilitation programs.

## 1. Introduction

The skeletal muscle is well known for the ability to adjust its physiological and mechanical characteristics to varying patterns of metabolic and mechanical stimuli induced by contractile activity [1,2]. Muscle stiffness or hardness, which may be simply defined as the resistance offered by muscles to compression, is a mechanical property that adapts in response to muscle use [3]. Unlike other tissues, skeletal muscle stiffness varies dynamically with muscle length changes and contraction status [4,5,6,7,8,9,10]. In addition to such transient changes, pathological modifications of the muscle tissue as a result of musculoskeletal disease, often affect its stiffness [11,12,13,14]. Repeated muscle use, including resistance training is also known to induce significant, albeit reversible, changes in muscle stiffness [15,16,17,18].

Quantitative assessment of muscle stiffness usually uses hardmeter devices or biomechanical methods to evaluate changes in muscle-tendon length-tension relationship. However, these techniques have important limitations, since they cannot separate changes in stiffness between different tissues or between layers of muscles. In this respect, imaging techniques, such as ultrasound quasi-static elastography (QSE), might allow measuring changes in muscle tissue stiffness with better spatial localization [19,20,21,22]. QSE is nowadays implemented in many commercial ultrasound (US) imaging machines, allowing real-time visualization of the amount of muscle tissue strain caused by repetitive light compressions applied directly onto the muscles with the US probe. While QSE was initially developed to enable an easier identification of abnormal structures on the basis of their higher stiffness relatively to the surrounding healthy tissue, it was soon applied for measuring muscle stiffness in active muscles [11]. With QSE, tissue strain magnitude caused by the compressive stresses is depicted as a colour-coded strain map (also termed “elastogram”), overlaid to the B-mode image, and semi-quantitative measurements of tissue stiffness may then be obtained by colour pixels counting [21,23].

In relaxed skeletal muscles, elastograms typically display a sparse colour pattern, characterized by scattered harder (blue, bluish colours) and softer (red, reddish colours) regions [11,21]. By comparing muscle elastograms’ colour patterns, QSE has been successfully used to study changes in skeletal muscle stiffness associated with musculoskeletal (MSK) disorders [11,12,24], including muscle spasticity [25], myofascial trigger points [14], muscle contractures associated with joint dysfunction [26], and congenital muscle dystrophy [24].

Together with its widespread use in the clinic, US elastography has also been used to study muscle coordination within complex muscle groups during movement [18,27,28]. Ultrasound elastography is useful in this case since it allows real-time measuring of muscle stiffness in different regions of the muscles (e.g., the muscle belly on the tendon), between superficial and deep muscles, and within complex multifunctional muscle groups, like in the neck [9] or in the shoulder girdle [27,29]. For example in the shoulder, US strain elastography allows one to describe the patterns of contraction of single muscles within an anatomical complex muscle group [27,29,30].

The above examples address applications of ultrasound QSE to pathological changes in muscle tissue stiffness or caused by contraction. Only few studies report the use of QSE for studying changes in relaxed muscle tissue stiffness in response to strength or endurance training [12,31]. To our knowledge, only one study has used US elastography (shear wave elastography) to investigate the impact of strength training on muscle stiffness, and concluded that six weeks of elbow flexors strength training could not change biceps brachii’s stiffness [32].

There are several studies that compare the influence of concentric versus eccentric training exercises on the adaptations induced in the knee extensor muscles [33,34,35]. In general, the results point to a clear improvement in the maximum levels of force production, in an isometric regime, without being able to identify an evident advantage of one mode of training over the other [34,35]. From a morphological point of view, studies suggest that the contraction regime is not a differentiating factor from the hypertrophic response to training, although they consider that the particular characteristics of the compared protocols must be safeguarded. However, Roig et al. argue that the increase in total strength, assessed through the sum of the moments of force produced in eccentric, isometric, and concentric regimes, is higher in eccentrically trained individuals [36]. Roig et al. also suggest that eccentric training is capable of promoting gains in muscle volume greater than those obtained with concentric training. Such adaptations are normally attributed to the greater mechanical overload imposed by eccentric contractions [37] and the high structural damage [38] caused by this mode of contraction. Likewise, there is also some controversy regarding the adaptive response of muscle architecture to both modes of contraction. Due to these factors we consider it important to evaluate and analyze the two types of training and their effects on muscle stiffness.

It is possible that six weeks of strength training were not sufficient to induce significant morphological and mechanical changes in the trained muscles, so the question remains whether strength training is able to alter muscle tissue stiffness and whether such change in stiffness can be detected by ultrasound QSE. In addition, it is known that muscle adaptation to strength training differs depending on the mode of contraction (i.e., concentric or eccentric) [34,39], velocity of contraction [29,40], or whether muscles contract in shortened or lengthened positions [41,42]. Therefore, in this study we also investigated whether the eventual change in muscle tissue stiffness was affected by the mode and amplitude of contraction after 15 weeks of isokinetic training of the knee extensors.

## 2. Methods

### 2.1. Participants

Twenty-eight young healthy males (mean ± SD; age: 20.0 ± 3.3 years, height: 1.75 ± 0.05 m, weight: 69.8 ± 6.7 kg) volunteered to participate in this study. Participants were fully informed of the purpose and procedures of the study and provided written informed consent. The study conformed to the guidelines of the Declaration of Helsinki and was approved by the ethics committee of the Faculty of Human Kinetics (CEFMH 13-2013). Anonymity and confidentiality of the collected data were assured. The study was conducted for scientific purposes only. All participants signed an informed consent prior to the study.

Participants were separated into a control group (Con) (*n* = 8) and a strength-training group (*n* = 20). Participants in the strength-training group were further separated in two groups according to the mode of contraction: one performed concentric training (GCon; *n* = 11), the other performed eccentric training (GEcc; *n* = 9). Participants with any injury of the lower limbs in the past six months, an orthopaedic condition or surgery involving the lower limbs were excluded. Groups were divided according to the availability of the participants. Participants in the control group were advised to avoid resistance training during the study.

### 2.2. Strength Training Program

**Figure 1:**
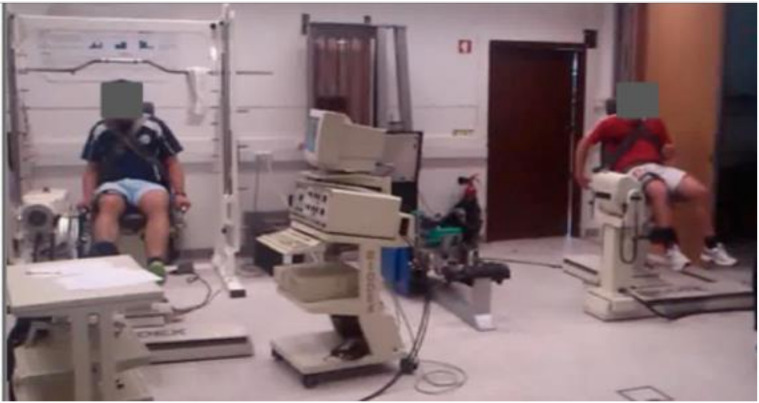
Isokinetic training session. Two participants were performing their training session using the Biodex 2 system (participant on the left) and Biodex 3 system (participant on the right).

Strength training consisted of three training sessions per week (training sessions were separated by one or two days rest) for 15 weeks, with a total of 45 training sessions [43,44]. All participants attended at least 42 out of the 45 planned training sessions (compliance rate = 93.3%). Strength training was done using isokinetic dynamometers (Biodex Medical Systems, Shirley, NY, USA) and consisted of maximal concentric (group GCon) and eccentric (group GEcc) contractions of knee extensors from both sides. Each limb was randomly selected to perform the contractions either along the full knee range of motion (ROM) allowed by the dynamometer (from 100 to 0 degrees of knee flexion) or along a restricted ROM (from 60° to 0°). Each training session began with a 10-min warm-up, consisting of cycling on a cycle ergometer (Monark Ergomedic 894E; Langley, WA, USA), with a workload of 75–80 W, or running on a treadmill (h/w/Cosmos e Pulsar 3p 4.0) at a self-selected velocity (8–12 km/h^−1^). The warm-up finished with 3–5 submaximal knee extension contractions performed on the isokinetic dynamometer. The concentric training was performed on a Biodex isokinetic dynamometer (Biodex System 3 or Biodex System 2; Biodex Medical Systems) using a pre-programmed isokinetic knee extension/flexion protocol (Figure 1). The number of sets, the number of repetitions within each set, the angular velocity, and the resting time between sets were defined in the protocol [44,45,46,47]. Participants were asked to perform the contractions as strong and as fast as possible and to keep their effort along the entire ROM. The eccentric training followed exactly the same protocol as that of concentric training.

During eccentric training, participants were also asked to perform eccentric contractions with their knee flexors, thus avoiding any concentric contractions with the knee extensor muscles, ensuring a purely eccentric training. The training program included five progression phases. During the first 3 weeks, the protocol included 5 sets of 6 isokinetic knee extensions at an angular velocity of 60°.s^−1^. To compensate for the shorter ROM, the number of contractions in each set was raised to ten in the legs training in this condition. During the following 12 weeks, the number of sets performed at 60°.s^−1^ decreased to only two, but additional sets were performed at 90°.s^−1^ (weeks 4–6), 120°.s^−1^ (weeks 7–9), 150°.s^−1^ (weeks 10–12), and 180°.s^−1^ (weeks 13–15). The number of sets performed at the higher angular velocities increased along the training weeks from five up to seven. For the limb training with restricted ROM, the number of contraction sets and repetitions were increased in order to match the total contraction time between the two legs [48,49].

### 2.3. Maximal Isometric Extension Torque

Knee maximal isometric extension torque was assessed on an isokinetic dynamometer (Biodex System 3, Biodex Medical Systems) before and after 15 weeks of strength training. The participants sat on the dynamometer chair and seat belts were fastened across the chest and pelvis. The leg was attached to the arm of the dynamometer by cushioned pads placed immediately above the lateral malleolus and hold in place with Velcro straps. The knee joint was carefully aligned with the rotating axis of the dynamometer arm. Maximal voluntary isometric contractions (MVC) of the knee extensors were repeated in 5° steps between 100° and 60° of knee flexion (complete knee extension: 0°) and in 10° steps between 60° and 30° knee flexion. Only contractions held for at least 2 s were accepted for further analysis. The sequence of the angles for the MVCs was selected randomly and the rest time between each MVC was of 2 min. The relatively short duration of the MVCs and the fact that only one repetition was performed for each knee angle was justified by the need to prevent muscle fatigue. However, a second MVC was done if the investigator or the participant himself considered the effort to be unsatisfactory or its duration was too short. The torque generated during the isometric MVCs was analog-to-digital converted at 16-bits resolution and at a 1 kHz sampling rate (MP100, Biopac, Santa Barbara, CA, USA). The maximum knee extension torque produced before and after the strength training period were then recorded and used for analysis [43,44].

### 2.4. Ultrasound Scanning

**Figure 2:**
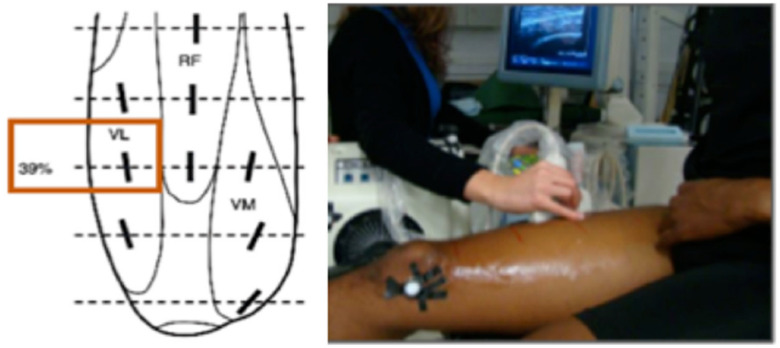
Diagram showing the location from where the ultrasound scans were collected (left). Picture of one participant sat on the dynamometer during ultrasound scanning (right).

Ultrasound scans were collected from the VL bilaterally at a region located at 39% of the distance between the upper edge of the patella and the anterior superior iliac spine (Figure 2) at baseline and after the strength training using a commercial US system (HITACHI EUB 7500, Hitachi Medical Corporation, Tokyo, Japan), equipped with a 7–12 MHz linear probe [50]. The US scans were acquired with participants seated on the isokinetic dynamometer, with 10° of knee flexion, and muscles fully relaxed [44]. The probe was placed aligned with the muscle fascicles (longitudinal scan). An experienced certified MSK ultrasound sonographer was responsible for collecting and analysing US data. To dismiss the possibility of circadian effects, the same person attended the laboratory for US data collection at approximately the same time of day before and after strength training.

Ultrasound settings were adjusted and optimized individually during the first session. The settings were then registered and implemented again during the second visit. The US gain was set at 25% of its range, dynamic range was maintained at 70 dB, time compensation was kept at neutral position, and depth was fixed at 65 mm for all scans. For strain elastography acquisition, light compressions were applied with the US probe at a frequency 3–4 Hz using the feedback provided by the manufacturer’s software and displayed on the screen. Elastography images were recorded in video sequences and in bitmap (BMP) format and stored in a computer hard disk. Two images were selected from each video for measurements of muscle stiffness.

### 2.5. Semi-Quantitative Elastography

**Figure 3:**
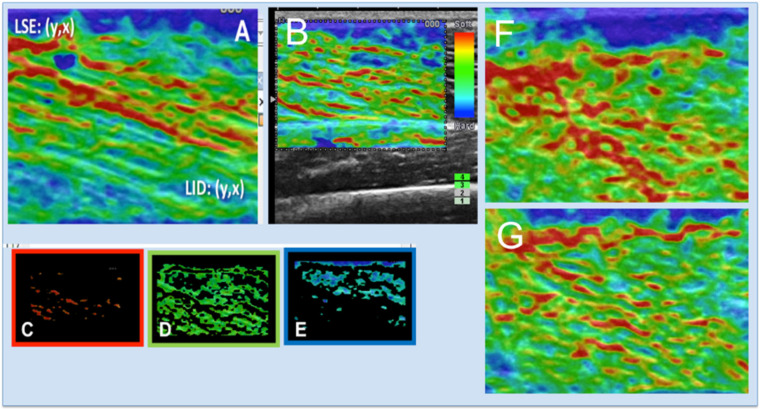
Strain elastography maps and red, green, and blue pixels counting. (**A**) Elastogram map displayed by the MATLAB routine. (**C**–**E**) Red, green, and blue pixels from A. (**B**) Elastogram superimposed to the B-mode image. (**F**) VL’s elastogram at baseline. (**G**) VL’s elastogram of the same participant as in (**F**) after resistance training (visible the decrease in the amount of red pixels compared to before strength training).

Muscle stiffness was assessed semi-quantitatively by the fraction of red, green, and blue pixels measured within a ROI using a routine written in MATLAB 20.0 software (The MathWorks, Inc., Natick, MA, USA) (Figure 3).

For each image, a rectangular ROI was drawn centred on the VL and with a minimum size of 5 × 7 mm. Tissue elasticity was represented by colour-coding [12]. Each pixel within the elastogram is assigned one of 256 specific colours depending on the amplitude of deformation, but three basic colours were used, called encoding RGB (red-green-blue). Colour ranged from red, corresponding to softer tissues, to blue, corresponding to harder tissues responding with less deformation to the applied pressure [12,51]. The green colour indicates tissues with medium deformation and lying between the red and blue coded tissues [51].

### 2.6. Statistical Analysis

In the control group, measures of knee torque and of VL elastography were collected only from the right leg. Therefore, statistical analysis for the effect of strength training was performed separately for the control group and the two strength-training groups. Pairwise comparisons were performed using t-test (control group). For the two strength-training groups, differences in knee maximal torque and in percentage of colour pixels before and after strength training were tested by mixed two-way analysis of variance (ANOVA) with a between-subjects factor with two levels (GCon and GEcc groups) and two within-subject factors, both with two levels (pre-and-post strength training and right and left leg). The sphericity assumption was assessed with Mauchly’s test and, if necessary, the Greenhouse-Geiser corrected significance levels were used. Correlations between elastography and muscle strength data were studied using linear regression and Pearson correlation coefficient. Partial eta square (η^2^) values were calculated to estimate effect sizes.

Intra-session (intra-observer) reliability for QSE values was assessed using intra-class correlation coefficient (ICC) (3.1; method: two-way mixed, consistency) and its 95% interval of confidence. SEM and SDC were also calculated. SEM indicates the precision of the measurement and was calculated based on the ICC and the SD of the mean of the differences between the two measurements (i.e., SEM = SD√1 − ICC). The SDC was based on the SEM, using the formula SDC = 1.96 × √2 × SEM. Data is reported as mean ± SD. Threshold for statistical significance was set at *p* < 0.05. All analyses were performed with SPSS 20.0 (SPSS Inc., Chicago, IL, USA) software package.

## 3. Results

Table 1 shows the data for maximal knee extensor torque before and after strength training. After strength training, maximal knee extensor torque increased significantly in both training groups [F_(1,18)_ = 58.583; *p* < 0.001; η^2^ = 0.874], but it did not change in the control group (*p* = 0.74). The increase in maximal knee extensor torque was similar in the GCon and GEcc groups as well as in the two limbs [pre-post vs. group interaction, F_(1,18)_ = 0.006; *p* = 0.938; pre-post vs. left-right interaction, F_(1,18)_ = 0.149; *p* = 0.704]. 

### Semi-Quantitative Elastography

Table 2 presents the mean fractions for red, green and blue pixels in VL’s elastograms for each group and before and after strength training. Regardless of the experimental group, leg, or time point, the fraction of red pixels in VL’s elastograms was consistently lower compared to the fraction of green and blue pixels. The percentage of red pixels relative to the total amount of pixels in each elastogram (Figure 3) varied in the range 8–16%. The percentage of green and blue pixels in each elastogram was comparable between each other, ranging between 26–38.7% (Table 2).

**Figure 4:**
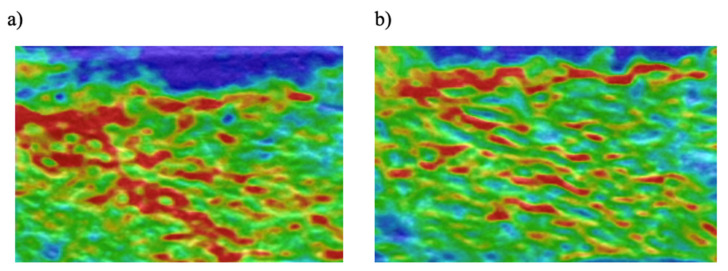
Examples of an elastogram collected from one participant in the strength-training group before training (**a**) and after training (**b**). Visible the typical ribbed elastic appearance of the elastogram from the healthy muscle tissue as well as the decrease in the amount of red colour and increase in green and blue colours after the strength training.

In the control group, the fraction of red and green pixels measured at the beginning and the end of the study remained unchanged. However, the fraction of blue pixels was increased at the end of the study (*p* < 0.01). Strength training produced significant changes in the fraction number of colour pixels in the elastogram of the VL (Figure 4). As the result of strength training there was a decrease in the fraction number of soft, red pixels [F_(1,18)_ = 25.490; *p* < 0.001; η^2^ = 0.586], and an increase in the fraction number of the harder green and blue pixels [pre vs. post strength training for green and blue pixels respectively: F_(1,18)_ = 17.179; *p* < 0.01; η^2^ = 0.488; F_(1,18)_ = 6.522; *p* < 0.05; η^2^ = 0.266]. These data are compatible with increased muscle stiffness as a result of strength training. Changes in the relative amounts of red, green and blue colour pixels after strength training were similar in GCon and GEcc groups and between the right and left legs. 

Before strength training, maximal knee extensor torque was positively correlated with the percentage of red pixels within the VL’s elastograms (r^2^ = 0.43; *p* < 0.01) and negatively correlated with the percentage of blue pixels (r^2^ = 0.29; *p* < 0.05). After training, no correlations between maximal knee extensor torque and number of red, green or blue colour pixels within VL’s relative strain maps could be found. 

Table 3 reports the reliability data regarding colour-mapping measures. Moderate intra-session ICCs were found. For all the three colours, ICC values were around the 0.6 acceptance level. 

SEM values for each of the three colours were similar, ranging between 0.04 and 0.06. SDC values varied between 0.10 (green colour for left thigh) and 0.16 (blue colour for both thighs) (Table 3). Blue colour tended to show higher absolute SEM and SDC values compared to red and green colours. Contrarily, SEM and SDC values for green colour were consistently smaller (Table 3).

Table 4 reports the ICCs for data recorded in the same session (intra-observer reliability). Moderate intra-observer ICCs were found. Only the measurements of green pixel fractions presented ICC values under the 0.6 acceptable level. 

## 4. Discussion

In this study we showed that 15 weeks of strength training increased the stiffness of the trained VL as assessed by ultrasound QSE. We also showed that the effect of strength training on muscle stiffness is similar whether training is performed in concentric or eccentric mode or if the muscle is made to contract through longer or shorter length ranges. To the best of our knowledge, this is the first time that increased muscle stiffness as a result of strength training is demonstrated by means of US elastography. 

Recently assessment of muscle hardness has received much attention as a relatively accessible way to evaluate acute muscle tissue changes as a result of repeated muscle activity [52]. Despite the limitations of US elastography, its feasibility as a means to assess passive muscle tissue stiffness has been demonstrated by several studies [13,19,53]. Colour-coded elastograms of healthy, relaxed skeletal muscles typically display complex and irregular colour patterns in which green and blue colours predominate, meaning that the stiffness of the skeletal muscle tissue is moderate but not strictly homogeneous [21,43,53]. Our study seems to confirm that VL’s stiffness is also heterogeneous, with patchy areas of red colour surrounded by areas of predominant green and blue colours in line with Drakonaky et al. who defined the muscle as “an inhomogeneous mosaic of intermediated or increased stiffness with scattered softer and harder areas” [21]. Presently, it is not known whether information can be extracted from the colour or grayscale patterns visible on strain US elastography images of skeletal muscles and whether such information could be associated with anatomical or physiological features. However, when viewed longitudinally, the few patchy areas with the same colour observed in colour-coded elastograms of skeletal muscles resemble the spatial arrangement of muscle fascicles, thus suggesting that a relationship between transverse strain behaviour and the skeletal muscle’s architecture may indeed exist. Regardless the apparent stochastic character of colour-coded skeletal muscle elastograms, their relative amounts of softer and harder colours change in a systematic way and in the expected direction (i.e., increased amount of harder colour pixels) when skeletal muscles contract or stretch [25]. Also, muscle tissue changes associated with congenital and traumatic myopathies are discernible with US strain elastography, which strengthens the validity and sensitivity of this imaging technique for assessing skeletal muscle mechanical properties [11,12,21,43,53].

By using strain US elastography, changes in muscle stiffness due to repeated contractile activity have been shown to occur [13,28,44,45,52,53]. After a series of forceful concentric and eccentric contractions of the elbow flexors, strain US elastography images showed increased strain resistance in biceps brachii in comparison to that of a constant-stiffness reference material placed in between the US probe and the skin [18]. Such changes in biceps brachii’s colour-coded elastograms were relatively short-lasting and were accompanied by an increase in muscle hardness, as measured with a tissue hardness meter [18]. The increased muscle stiffness occurring after intense muscle contractions has been explained on the basis of increased hydrostatic pressure, raised muscle blood flow, and elevated intramuscular fluid content, particularly within the extracellular space [46,47]. Other possibility, which might also underlie the changes in muscles fluid content, is the occurrence of small structural changes or injuries, which might trigger an inflammatory response, altering the stiffness of the muscle regions affected or even leading to increased residual muscle contraction [28,44,49]. However, it is unlikely that raised intramuscular pressure and exercise-induced muscle oedema could explain the changes in VL’s stiffness that were observed in the present study as a result of strength training. In our study, participants were tested few days after having performed the last training session and they were carefully instructed to keep their knee extensors relaxed during US scanning. Therefore, the increase in relative strain seen in the US elastograms of strength trained VL is likely related with lasting and adaptive changes in the mechanical properties of the muscle tissue, leading to an increase in its stiffness. 

Increased muscle stiffness as a result of strength training has been demonstrated in several occasions [2,16,50,51]. Muscle stiffness, defined as the relationship between the change in muscle length and the amount of stress applied to the muscle, is generally regarded as the strain resistance along the muscles’ longitudinal axis or the resistance to muscle elongation and is given by the steepness of the passive or active length-tension curve of the muscle-tendon complex. The compressive force that is applied onto the muscle tissue during strain US elastography strains the underlying tissues in proportion to their stiffness, which is then derived by contrasting the US echoes during compressed and uncompressed conditions [21]. Changes in joints’ resting position and tendon stiffness after intense muscle contractile activity and strength training are a further indication of increased muscle hardness and stiffness associated with increased muscle use [52]. Taş et al. (2017) showed that the quadriceps femoris strength was positively correlated with patellar tendon stiffness and thickness [44]. After two months of resistance training of the elbow flexors, the resting elbow position significantly moved towards increased flexion, while the elbow joint’s stiffness, a parameter that informs about the biomechanical properties of the overall structures making up this joint (i.e., joint surfaces, cartilages, joint capsule and ligaments), including the several muscle-tendon complexes crossing the joint, significantly increased [16]. Increased gastrocnemius muscles stiffness was also reported after plyometric training of the human ankle plantar flexor muscles, a kind of training in which cycles of powerful lengthening and shortening muscle contractions are performed, thus resembling the behaviour of ankle plantar flexors during running or jumping [2].

There are several possible mechanisms underlying the increase in muscle stiffness seen after strength training. Changes in muscle architecture and muscle hypertrophy might be responsible for increased muscle passive stiffness [3,51]. A significant positive correlation between biceps brachii and brachial anterior volumes and elbow joint stiffness has been reported [51]. Nonetheless, differences in muscle stiffness in response to axial compression and to elongation might exist. Muscle hardness is the term that is usually employed to name the palpable resistance of the muscle tissue to an applied pressure and, in this sense, is closer to strain elastography qualitative or semi-quantitative measures than the biomechanical muscle-tendon complex elongation apparent stiffness. However, muscle hardness and muscle elongation stiffness are correlated and, therefore, a rise in muscle hardness occurs when there is an increase in muscle’s tension caused by contraction [3,52] or by passive stretch [3].

In the present study, we had different groups undertaking concentric and eccentric strength training. Also, in one of the limbs muscle contractions were performed along a larger movement amplitude of the knee joint (i.e., knee extensors trained in shortened length). However, strength increase was similar in the two strength-training groups and in both limbs, showing that the efficacy of the strength training, in terms of the gain in maximal voluntary isometric strength, was similar irrespectively of the kind of muscle contraction employed (i.e., Conc vs. Ecc) and the amount of active shortening or lengthening (full ROM vs. limited ROM). Likewise, the percentage changes in “softer” and “harder” colours in US elastograms of the VL after strength training was similar amongst the different training conditions. Previous studies show that the increase in human elbow flexors stiffness is related with the total amount of muscle work during training [16]. Whether such discrepancies between our results and those of others reflect fundamental differences between elbow flexors and the VL or differences in the training and testing protocols between the two studies is unknown at this point. 

It is possible that the similar changes in the US elastograms between the different strength training conditions may be associated with the limited precision of QSE measures. Previously, Muraki et al. and Yanagisawa et al. showed very high ICC values (around 0.9) for the strain ratio in the supraspinatus and biceps brachii muscles [18,29]. However, large variations in ICC values (range 0.6–0.9) for US elastography measures collected from the gastrocnemius muscle evaluation have also been reported [53]. Our study showed a moderate (ICC: 0.6–0.7) intra-evaluation reliability for colour mapping values by ultrasound QSE. The modest reliability values found for our measures of the amount of colour pixels could be due to the compression operator-dependence of the measurements [5,43,49,50], and the fact that the scans were collected with participants seated on the dynamometer, maybe a less restricted and controlled posture, compared with other studies [48,49,50]. The limited reliability of ultrasound QSE and relatively large SEM and SDC values might explain why there was a significant change in the amount of green pixels between the beginning and the end of the study in the control group. The moderate reliability of ultrasound QSE should be taken into consideration when applying this method for the study of physiological adaptations of the skeletal muscle, and strengthens the necessity of following a strict protocol for ultrasound and data collection.

### 4.1. Limitations

Strain US elastography offers qualitative information of tissue strain behaviour by direct viewing of a grey-scale or colour-coded image superimposed on the B-mode image. However, the range of strains is dynamically adjusted depending on the difference between the lowest and the highest strained tissue within the scanned region, thus rendering the elastogram a relative image only. To make the qualitative colour-coded information of QSE a semi-quantitative measure, ratios of relative strains between the ROI and a reference can be employed. The overlying subcutaneous fat can be used as reference, but it is not possible to guarantee that the subcutaneous fat properties remain unchanged after the training. Moreover, the thickness of the subcutaneous fat may be too small to allow selecting a ROI. This limitation is particularly relevant when studying young and very active subjects, as in this study, who have a very low percentage of body fat. As an alternative, a material of homogeneous mechanical properties might be used for reference [18,21]. However, our elastographic measures were not normalized to a reference. Therefore, we should emphasize that there is a small chance that the increase in the area fraction occupied by the harder green and blue colours that was noted in our study after strength training might not reflect heightened muscle hardness. Indeed, structural changes could have altered the relative hardness of the different tissues that are scanned and thus modified the colour distribution within the elastogram. However, increased fraction of “harder” colour in the VL elastograms would have happened only if some tissue harder than the muscle prior training had turned softer by the end of the study, which is unlikely. Another limitation of this study relies on the operator-dependence of QSE. Although this limitation cannot be completely avoided, it was minimised by having the same operator conducting every US scan and by carefully selecting images for analysis obtained when compression frequency stood in the range 3–4 Hz.

### 4.2. Practical Applications

The use of ultrasound elastography can be a useful tool in monitoring athletes’ performance and verifying muscle changes that result from the practice of physical exercise. It will be an added value to add this exam to the athletes’ routine medical evaluation, since the results show that morphological and physiological changes appear, not pathological at the muscular level, and that are also not detected in the current routine tests. Through ultrasound we can see what kind of morphological and physiological effects a given training program can have at the muscle level.

## 5. Conclusions

This study offers the first demonstration that after 15 weeks of strength training there is an increase in the stiffness of the relaxed VL that can be measured with strain US elastography. This study reports data of VL’s stiffness obtained by QSE and the results show increased stiffness as a result of strength training, although without differences between different modes of muscle contraction. Although several studies already showed the viability of the QSE to assess muscle stiffness, this study is the first one to demonstrate an increase in muscle stiffness as a result of strength training by means of ultrasound elastography. While ultrasound QSE has some limitations, it is still employed to assess muscle function in several contexts also because it is one of the first ultrasound elastography methods implemented in commercial equipment.

## Figures and Tables

**Table 1:** Data for maximal isometric torque produced by the knee extensors before and after strength training for the right and left limbs.

Maximal Voluntary Isometric Torque (Nm)				
	Pre-Training		Post-Training	
Group	Right Limb	Left Limb	Right Limb	Left Limb
Control	303.5 ± 30.8		300.0 ± 35.7	
GCon	300.3 ± 55.2	284.1 ± 46.7	377.3 ± 54.6 ***	374.3 ± 54.6 ***
GEcc	241.9 ± 35.0	229.2 ± 12.5	328.9 ± 42.8 ***	328.9 ± 42.8 ***

Data presented as mean ± SD. *** Significantly different from Pre-training based on ANOVA (*p* < 0.001).

**Table 2:** Data for colour pixels in vastus lateralis’ elastograms.

	Fraction of Colour Pixels (a.u.)				
		Pre-Training		Post-Training	
Group	Colour	Right Side	Left Side	Right Side	Left Side
**Control**	Red	0.156 ± 0.058		0.144 ± 0.065	
	Blue	0.297 ± 0.043		0.325 ± 0.079	
	Green	0.261 ± 0.067		0.333 ± 0.079 **	
**GCon**	Red	0.160 ± 0.050	0.111 ± 0.052	0.082 ± 0.023 ***	0.088 ± 0.027 ***
	Blue	0.295 ± 0.037	0.319 ± 0.043	0.341 ± 0.039 **	0.354 ± 0.041 **
	Green	0.290 ± 0.090	0.354 ± 0.095	0.381 ± 0.076 *	0.359 ± 0.063 *
**GEcc**	Red	0.126 ± 0.056	0.131 ± 0.063	0.092 ± 0.020 ***	0.089 ± 0.021 ***
	Blue	0.311 ± 0.048	0.284 ± 0.036	0.348 ± 0.063 **	0.313 ± 0.073 **
	Green	0.344 ± 0.096	0.368 ± 0.010	0.357 ± 0.069 *	0.387 ± 0.097 *

Data presented as mean ± SD. Significantly different from Pre-training: * *p* < 0.05; ** *p* < 0.01; *** *p* < 0.001.

**Table 3:** Data of Colour mapping for vastus lateralis and intra-class correlation coefficient (*n* = 8).

Colour Maping for Vastus Laterallis	1st Image Mean Fraction	ICC Consistency	SEM	SDC
Right Side				
Red colour	0.15 ± 0.07	0.59	0.05	0.13
Blue colour	0.30 ± 0.11	0.77	0.06	0.16
Green colour	0.30 ± 0.05	0.53	0.04	0.11
Total	0.75			
				
Left Side				
Red colour	0.12 ± 0.07	0.67	0.04	0.12
Blue colour	0.35 ± 0.10	0.68	0.06	0.16
Green colour	0.31 ± 0.05	0.63	0.04	0.10
Total	0.78			

Data presented as mean ± SD. ICC = intra-class correlation coefficient; SEM = Standard error of measurement; SDC = smallest detectable change.

**Table 4:** Data of Colour mapping for vastus lateralis and inter-class correlation coefficient (*n* = 7).

Group	Colour Mapping for Vastus Lateralis	1st Session Mean Fraction	2st Session Mean Fraction	ICC Consistency
**Control**	Red colour	0.15 ± 0.05	0.11 ± 0.02	0.44
	Blue colour	0.25 ± 0.08	0.34 ± 0.11	0.75
	Green colour	0.32 ± 0.07	0.33 ± 0.07	0.60

Data presented as mean ± SD. ICC = intra-class correlation coefficient.
